# Out of Sync: Parent-Child Behavioral Synchrony in Conduct Problem Subgroups

**DOI:** 10.1007/s10802-026-01491-6

**Published:** 2026-07-30

**Authors:** Silvana Kaouar, Georgette E. Fleming, Bryan Neo, Campbell McDonogh, Lucy Koh, Amanda B. Chan, Lily Chen, Sasha Forrest, David J. Hawes, Valsamma Eapen, Eva R. Kimonis

**Affiliations:** 1 Parent-Child Research Clinic, School of Psychology, The University of New South Wales, Sydney, NSW Australia; 2 School of Psychological Sciences, Macquarie University, Sydney, NSW Australia; 3 School of Psychology, The University of Sydney, Sydney, NSW Australia; 4 School of Psychiatry, The University of New South Wales, Sydney, NSW Australia

**Keywords:** Synchrony, Conduct problems, Callous-unemotional traits, Callous-unemotional variants, Parent training

## Abstract

Parent-child behavioral synchrony—the dyad’s moment-to-moment coordination of behavior and emotion—supports children’s socioemotional development, yet most evidence comes from cross-sectional community samples using global indices, limiting clinical insight for heterogeneous conduct problems (CP). This prospective study tested baseline differences and treatment-related change in discrete synchrony facets among clinic-referred children with CP, comparing CP-only, primary callous-unemotional (CU; CU without internalizing problems), and secondary CU (CU with internalizing problems) variants. Mother-child and father-child dyads of *N* = 234 children aged 2–7 years (74% boys) completed standardized play tasks that were coded into four Mutually Responsive Orientation dimensions (Coordinated Routines, Mutual Cooperation, Harmonious Communication, Emotional Ambience); parents completed questionnaires assessing child CP, CU traits, and internalizing symptoms. A subsample (*n* = 142) received parent training and was re-assessed post-treatment and at three-month follow-up. At baseline, mother-child Harmonious Communication and Emotional Ambience were significantly lower in secondary CU than in primary CU and CP-only groups. Parent training significantly improved synchrony across dyads from baseline to post-treatment, with maintenance for mothers. However, despite overall gains, synchrony remained lower for secondary CU than primary CU variants at follow-up (mothers), and father-child Harmonious Communication deteriorated from post-treatment to follow-up in secondary CU relative to improvement in primary CU. Findings indicate variant-specific synchrony deficits, especially affective facets, in secondary CU and attenuated normalization following parent training, refining etiological accounts and highlighting the need to tailor synchrony-focused treatment targets for CP subgroups.

**Supplementary Information:**

The online version contains supplementary material available at 10.1007/s10802-026-01491-6.

The parent-child relationship is a foundational context for children’s social, emotional, behavioral, and moral development. Supportive relationships are consistently associated with secure parent-child attachment, stronger self-regulation, and adaptive behavior (Ainsworth et al., 1978; Davis et al., 2017; Sfeir et al., 2026). A central process in these relationships is parent-child behavioral synchrony (“parent-child synchrony”): the dyad’s timely, reciprocal coordination of behavior and emotion during shared interactions, typically assessed via observed tasks (Ainsworth et al., 1971). Synchrony reflects ongoing, dynamic attunement—each partner orients and responds to the other's actions and affect in ways that are harmonious and meet each other’s needs. In infancy, caregivers largely scaffold this back-and-forth exchange; over time, children become increasingly active partners. Classic work has operationalized synchrony as timely maternal sensitivity to infant cues (e.g., fussing/crying infant met with soothing), with asynchrony marked by misinterpretation or neglect of infant signals (Ainsworth et al., 1978; Isabella et al., 1989; Tesson et al., 2023). Beyond infancy, smooth, connected exchanges, implicit shared expectations, and openness to mutual influence are salient markers (Aksan et al., 2006).

Child characteristics can facilitate or disrupt synchrony. Conduct problems (CP) are bidirectionally linked with fewer warm and more coercive interactions (Feldman et al., 2013; Hogye et al., 2023; Scaramella & Leve, 2004). Less empirical attention has been paid to temperamental features that may further impede synchrony, including callous-unemotional (CU) traits (i.e., lack of remorse/guilt, callous-lack of empathy). We therefore examined whether synchrony differs between established CP subtypes in clinic-referred children and whether treatment improves synchrony (Craig et al., 2021, 2023; Fleming et al., 2023; Frick et al., 2014).

## Parent-Child Synchrony and Conduct Problems

In community samples, higher synchrony prospectively predicts improved developmental and psychosocial functioning in toddlers and preschoolers at-risk for CP (Healey et al., 2010; Skuban et al., 2006), whereas lower synchrony and reduced positive affect relate to higher CP (Deater-Deckard et al., 2004; Sfeir et al., 2026). Scant research has examined synchrony in clinical samples but suggest similar patterns (Ambrose & Menna, 2013; Birk et al., 2022): clinic-referred children with CP show lower mother-child synchrony than non-clinical controls (Im-Bolter et al., 2014; Woltering et al., 2015), and higher baseline synchrony predicts greater reductions in child emotional lability, aggression, and CP following treatment (De Rubeis & Granic, 2012; Miller-Slough et al., 2015). Notably, synchrony quality matters: coordinated negative affect, escalating arguing and anger, and coercive “in-sync” patterns constitute maladaptive synchrony that can reinforce CP and ineffective parenting (Harrist & Waugh, 2002; Patterson et al., 1989; Sfeir et al., 2026), whereas reciprocal positivity and warmth are associated with adaptive outcomes (Deater-Deckard et al., 2004; Harrist & Waugh, 2002; Morgan et al., 2023).

## Parent-Child Synchrony and CU Traits

Despite developmental models positing a role for synchrony (Kochanska et al., 2005), few studies have tested links between synchrony and CU traits, especially in clinical samples (Frick et al., 2014). Multiple mechanisms predict impairment. First, earlier starting, more severe, persistent CP in CU elevations may entrench coercive cycles (Deater-Deckard et al., 2004; Hogye et al., 2023; Patterson et al., 1989). Second, fearlessness and punishment insensitivity reduce responsiveness to socialization (Fanti et al., 2024; Kochanska et al., 2015). Third, heightened parenting stress lowers parental responsiveness (Fanti & Centifanti, 2014; Fanti et al., 2017; Ribas et al., 2024). Fourth, reduced eye contact, emotion recognition, and empathy constrain affective coordination (Chan et al., 2023; Ciucci et al., 2024; Martinez-Cedillo et al., 2026). Lastly, lower levels of parental warmth—an environmental correlate of CU traits—may dampen shared enjoyment (Chau et al., 2025; Henery et al., 2025; Waller et al., 2018). Consistent with these mechanisms, community studies link higher CU traits to lower mother-child and father-child synchrony and show that higher mother-child synchrony predicts later decreases in CP among young children with elevated CU traits (Kochanska et al., 2013, 2015). Replication in clinical samples is lacking.

## Primary and Secondary CU Variants

*Within* CU presentations, subtyping by comorbid internalizing symptoms distinguishes primary and secondary CU variants (Craig et al., 2021). Secondary CU traits (high CU, high internalizing) are theorized to follow early-life exposure to adverse interpersonal contexts (e.g., violence, maltreatment) and involve heightened emotional reactivity (Kimonis, 2023), lower maternal responsivity, and more conflictual parent-child relationships (Kok et al., 2013; Li et al., 2023; Owens et al., 1998; van den Boom & Hoeksma, 1994). Mothers of children with secondary CU traits express fewer warm and more critical cognitions about their child relative to their primary CU counterparts (high CU, low internalizing; Kaouar et al., 2023), which relate to more hostile, coercive, and less sensitive parenting (Chau et al., 2025; Johnston et al., 2018; Leerkes et al., 2015). This profile plausibly predicts especially low synchrony, yet studies rarely disaggregate CU variants, leaving unclear whether synchrony deficits are driven by secondary CU traits. Examining synchrony for children with secondary CU traits helps shift the field beyond retrospective or broad indices of adversity, toward specific, observable, and clinically actionable interaction patterns that may be particularly relevant in this early childhood period.

## Synchrony Components

Beyond “total” synchrony, the *Mutually Responsive Orientation* scale (MRO; Aksan et al., 2006) for qualitatively coding synchrony from observed parent-child interactions, captures four dimensions (see Supplemental Table 2): behavioral or ‘action-based’ dimensions of Coordinated Routines (e.g., shared procedural expectations, task follow-through) and Mutual Cooperation (e.g., accepting roles, following each other’s lead), as well as emotional dimensions of Harmonious Communication (e.g., flowing conversation, connectedness) and Emotional Ambience (e.g., positive affect, enjoyment). It is plausible that these distinct synchrony dimensions relate differentially to CP subtypes. In CU traits, the behavioral dysregulation, reduced inhibition, emotion processing difficulties, and parental stress may impair both the behavioral and emotional aspects of dyadic synchrony, compared to children with CP-only. For secondary CU traits, heightened emotional sensitivity and more severe behavioral profiles may particularly undermine Emotional Ambience and Mutual Cooperation. Identifying which facets are compromised across CP subgroups can refine mechanistic treatment targets in early childhood.

## Treatment-Related Change

Parent-child synchrony is integral to conscience development, socialization, and empathy (Kochanska et al., 2005)—domains that are impaired in both primary and secondary CU traits (Craig et al., 2021, 2024). Parent-Child Interaction Therapy (PCIT; Eyberg & Funderburk, 2011) is a parent training intervention that directly coaches responsive, consistent, and predictable parenting—first strengthening enjoyment and warmth, then teaching safe, consistent discipline. PCIT robustly reduces child CP (Calderone et al., 2025; Lieneman et al., 2019). Preliminary studies show synchrony gains following PCIT-based or sensitivity-focused interventions (Crotwell et al., 2013; Ravn et al., 2011), but samples were not stratified by CU traits and largely excluded father-child dyads. Whether synchrony improves equally across CU variants remains unknown.

## The Current Study

We addressed these gaps in a clinical sample of young children with heterogeneous CP by testing baseline differences and treatment-related change in mother-child and father-child synchrony across CP-only, primary CU, and secondary CU variants. Guided by prior theory and evidence (Kimonis, 2023), we hypothesized: (1) lower synchrony for children with CU traits compared with CP-only across dimensions, with the lowest levels for secondary CU variants, particularly for Emotional Ambience and Mutual Cooperation; and (2) significant synchrony gains during PCIT and maintained at three months, with potential attenuation for secondary CU variants. We also examined group differences in change magnitude and in post-treatment and follow-up synchrony levels.

## Method

### Participants

Written informed consent was obtained from all participating parents. Participants were families drawn from across five clinical research studies conducted in Sydney, Australia, at either a community or university clinic providing clinical services to families of children referred for CP (*N* = 234; see Supplemental Table 1). Two studies oversampled children with CU traits for clinical research trials testing the efficacy of an enhanced version of PCIT for this subpopulation (Fleming & Kimonis, 2018; Kimonis et al., 2019). The primary referral reason for all families was child CP, but children varied on levels of CU traits. The final sample included *N* = 234 young children (*M* = 4.50 years, *SD* = 1.50, 74% boys) referred to the university clinic (*n* = 175, *M* = 4.99 years, *SD* = 1.39, 75% boys) or community clinic (*n* = 59, *M* = 3.04 years, *SD* = 0.64, 73% boys), and their parent(s) (232 mothers, 146 fathers). Only the university clinic subsample was assessed longitudinally (see Supplemental Fig. 1 for participant flow chart).

At the university clinic, the median annual household income was $141,000 (ranging AUD$18,000-$1,000,000; not reported by 19 families), and parents self-identified as Caucasian/White (73.1% mothers, 74.9% fathers), Asian (12% mothers, 7.4% fathers), Pacific Islander (1.1% fathers), Middle Eastern (4% mothers, 5.1% fathers), Aboriginal or Torres Strait Islander (0.6% mothers, 0.6% fathers), African (0.6% mothers, 0.6% fathers), or “Other” (8.6% mothers, 8.6% fathers; not reported by 5 parents). Income and ethnicity data were not collected by the community clinic and are unavailable, however the median annual household income in the clinic’s local government area was $72,280 (ABS, 2021).

Children were classified into three CP subgroups based on resolved scores (i.e., summing the highest item rating across reporters) on the 24-item *Inventory of Callous-Unemotional Traits - Preschool Version* (Kimonis et al., 2016), and the Internalizing composite scale of the *Achenbach System of Empirically Based Assessment - Child Behavior Checklist* (ASEBA CBCL; Achenbach & Rescorla, 2000, 2001). Final group classifications were: CP-only (*n* = 74), primary CU (*n* = 58), and secondary CU (*n* = 102; see Supplemental Materials for further group classification details, and comparisons on main study variables).

### Procedure

Parents completed main study questionnaires via hard copy or online Qualtrics survey platform. Each parent-child dyad separately engaged in a series of three, five-minute standardized parent-child interaction tasks including a child-led play, parent-led play, and clean-up situation. The assessor observed the play from behind a one-way mirror, providing verbal instructions to the parent via a wireless earpiece. All play interactions were videorecorded and later coded to measure parent-child behavioral synchrony.

For the university clinic subsample that was assessed longitudinally, families received either standard PCIT or its adaptation for children with CU traits (PCIT-CU; Fleming & Kimonis, 2018), determined through either random allocation or matching the treatment version to the child’s level of CU traits (i.e., standard PCIT for children low on CU traits, PCIT-CU for children high on CU traits). Consequently, longitudinal analyses controlled for the treatment version received.

This longitudinal subsample repeated assessment procedures at post-treatment (*n* = 93 mothers, *n* = 59 fathers), and at a three-month follow-up (*n* = 80 mothers, *n* = 55 fathers). During COVID-19 lockdowns, families completed their assessments online at home via a secure teleconferencing platform (*n* = 26 at baseline, *n* = 15 post-treatment, *n* = 9 follow-up).

Aim one of the present study was tested cross-sectionally using baseline data from the full sample (*N* = 234). Aim two was tested using baseline, post-treatment, and three-month follow-up assessment data completed by the longitudinal subsample (*n* = 175).

### Measures

All parent-report measures were completed separately by mothers and fathers, where possible. Resolved scores were computed for measures about the child. Tables 1 and 2 and present descriptives and Cronbach’s alphas. See Supplemental Materials for expanded details of present study measures.

#### Conduct Problems

Child CP was assessed using the Intensity scale of the *Eyberg Child Behavior Inventory* (ECBI; Eyberg & Pincus, 1999). The ECBI is a 36-item parent-rated measure of child CP (e.g., “Has temper tantrums”). In the current study, parents rated the frequency of the child’s problem behaviors on a 7-point scale, from 1 (never) to 7 (always), with resolved scores summed to compute total Intensity scale scores. Intensity scale scores range between 36 and 252 and have demonstrated excellent internal consistency (Cronbach’s α = 0.95; Eyberg & Pincus, 1999). In the present study, ECBI Intensity scores also demonstrated excellent internal consistency.

#### Callous-Unemotional Traits

CU traits were assessed using the 24-item total score of the 24-item parent-rated ICU (e.g., “Feels bad or guilty when he/she has done something wrong”). In the current study, parents rated the applicability of each item for their child on a 4-point scale, from 0 (not at all true) to 3 (definitely true), with resolved scores summed to compute an ICU total score. ICU total scores range between 0 and 72 and have demonstrated acceptable internal consistency (Deng et al., 2019; Kimonis et al., 2016). In the present study, total ICU scores showed good internal consistency.

#### Internalizing Symptoms

Child internalizing symptoms were assessed using parent-reported *T*-scores on the 36-item Internalizing composite scale of the age-appropriate version of the ASEBA CBCL (e.g., “Too fearful or anxious”; Achenbach & Rescorla, 2000, 2001). In the current study, parents rated the applicability of each item for their child on a 3-point scale, from 0 (not true) to 2 (very true or often true), with resolved scores summed to compute the composite score. Composite scores were then converted into *T-*scores, which range from < 50–100. ASBEA CBCL1.5-5/6–18 Internalizing composite scale scores have demonstrated excellent internal consistency (Achenbach & Rescorla, 2000, 2001). In the present study, the Internalizing composite scale had good internal consistency for both 1.5-5 and 6-18-year-old age versions.

#### Parent-Child Behavioral Synchrony

Synchrony was coded from video recordings of parent-child interactions using the *Mutually Responsive Orientation* scale (MRO; Aksan et al., 2006). MRO comprises a Total synchrony score and four synchrony dimensions: Coordinated Routines, Harmonious Communication, Mutual Cooperation, and Emotional Ambience (see Supplemental Table 2). Only MRO synchrony dimension scores were used in this study. Results for Total synchrony scores are available upon request. Since we aimed to assess treatment-related change in MRO, we used a wider range of numerical anchor points than is typically used for ratings of the four synchrony dimensions (range: 1–3), to increase variability in scores. Each five-minute play scenario was coded using MRO, and resultant scores were averaged across the three play scenarios, creating the overarching synchrony dimension scores that were used in analyses. Coders (*k* = 5) were masked to research trial, assessment timepoint, and child CP group classification. For the present study, a randomly selected subset of 15% of baseline assessment videos were double-coded to calculate inter-rater reliability using intra-class correlations (ICCs). ICCs were acceptable for Harmonious Communication (0.73) and Emotional Ambience (0.70), and good for Coordinated Routines (0.77) and Mutual Cooperation (0.89).

## Results

### Correlations Between Main Study Variables

All analyses were conducted in SPSS version 29. Data were screened for outliers and assumptions tested (see Supplemental Materials). Tables 1 and 2 present descriptive statistics and Pearson’s correlations for mother-child and father-child behavioral synchrony scores, and parent-report measures. Synchrony dimension scores were significantly positively inter-correlated, for both mothers and fathers. Mothers’ synchrony scores were also significantly positively correlated with fathers’ synchrony scores across dimensions. However, scores on father-child Emotional Ambience were uncorrelated with mothers’ synchrony scores, except for a significant positive correlation with mother-child Mutual Cooperation. Also, scores on father-child Harmonious Communication were only significantly positively correlated with mother-child Harmonious Communication and Mutual Cooperation, but not mothers’ Coordinated Routines or Emotional Ambience. 

In terms of child factors, only mother-child Harmonious Communication and Emotional Ambience scores were significantly negatively correlated with ICU scores^1^. For mothers and fathers, synchrony scores were otherwise uncorrelated with ECBI, ICU, or CBCL Internalizing scores.

**Table 1:** Descriptives and correlations among mother-child and father-child behascripvioral synchrony scores at basline

Study Variables	1.	2.	3.	4.	5.	6.	7.	8.
1. M Coordinated Routines	1							
2. M Harmonious Communication	0.59**	1						
3. M Mutual Cooperation	0.81**	0.73**	1					
4. M Emotional Ambience	0.62**	0.80**	0.70**	1				
5. F Coordinated Routines	0.32**	0.26**	0.28**	0.22*	1			
6. F Harmonious Communication	0.14	0.23*	0.22*	0.11	0.66**	1		
7. F Mutual Cooperation	0.31**	0.24*	0.35**	0.23*	0.82**	0.72**	1	
8. F Emotional Ambience	0.13	0.18	0.23*	0.19	0.63**	0.78**	0.75**	1
Mean	6.75	6.16	6.71	6.80	6.82	6.00	6.83	6.70
*SD*	1.74	1.63	1.67	1.56	1.71	1.42	1.55	1.55
Skewness	-0.41	0.08	-0.41	-0.28	-0.60	-0.12	-0.62	-0.70
Kurtosis	-0.75	-0.73	-0.69	-0.63	-0.56	-0.24	-0.01	0.01

Note. M = Mother; F = Father. Synchrony dimension scores mean summed across the three play scenarios from which they were coded

*p < .05. **p < .01

**Table 2:** Descriptives and correlations between baseline mother-child and father-child synchrony scores and parent-reported child measures

Study Variables	ECBI Intensity	ICU	CBCL INT
M Coordinated Routines	0.01	0.04	− 0.08
M Harmonious Communication	− 0.10	− 0.16*	− 0.07
M Mutual Cooperation	− 0.10	− 0.09	− 0.13
M Emotional Ambience	− 0.11	− 0.16*	− 0.07
F Coordinated Routines	0.06	0.05	− 0.08
F Harmonious Communication	− 0.03	− 0.09	− 0.09
F Mutual Cooperation	− 0.04	− 0.05	− 0.15
F Emotional Ambience	− 0.10	− 0.08	− 0.19
ECBI Intensity		0.61**	0.40**
ICU			0.39**
Mean	168.27	33.26	64.59
*SD*	31.38	10.72	9.62
Skewness	-0.69	0.00	-0.44
Kurtosis	0.81	0.67	0.25
Cronbach’s α	0.91	0.89	0.89, 0.86

Note. M = Mother; F = Father; ECBI Intensity = Eyberg Child Behavior Inventory Intensity scale; ICU = Inventory of Callous-Unemotional Traits; CBCL INT = Child Behavior Checklist Internalizing. Cronbach’s alphas for CBCL INT presented for younger and older age versions, respectively

*p < .05. **p < .01

### Aim One: Subgroup Differences in Baseline Parent-Child Behavioral Synchrony

To test aim one, we used generalized linear models (GLM) with Bonferroni-corrected planned comparisons between the three CP subgroups (presented *p*-values are Bonferroni-adjusted). Child CP severity and age were included as covariates (see Supplemental Materials for analyses additionally controlling for child Attention-Deficit/Hyperactivity Disorder (ADHD) symptoms). First, we compared children high versus low on CU traits. Mother-child dyads in the two groups did not differ in their Coordinated Routines (*M*diff = -0.51, *p* = .074, 95% CI [-1.08, 0.05], *d* = 1.79) or Mutual Cooperation scores (*M*diff = -0.44, *p* = .111, 95% CI [-0.99, 0.10], *d* = 1.59). However, children with CP + CU had significantly lower mother-child Harmonious Communication (*M*diff = -0.65, *p* = .018, 95% CI [-1.18, -0.11], *d* = 2.37) and Emotional Ambience (*M*diff = -0.70, *p* = .007, 95% CI [-1.21, -0.19], *d* = 2.69) than children with CP-only, when controlling for CP intensity. There were no significant differences in father-child synchrony scores between children with high versus low CU traits.

Supplemental Table 3 presents results of GLM tests of model effects with planned comparisons between all three CP subgroups on parent-child synchrony dimensions. Mother-child interactions for the secondary CU group were characterized by significantly lower Harmonious Communication (*M*diff = -0.74, *p* = .038, 95% CI [-1.46, -0.03], *d* = 2.50) and Emotional Ambience (*M*diff = -0.72, *p* = .035, 95% CI [-1.40, -0.04], *d* = 2.52), than the CP-only group. There were no other group differences for the remaining mother-child synchrony dimensions. For father-child dyads, there were no significant differences between CP subgroups on any synchrony dimension.

In light of our findings that parent-child synchrony was lower for children with secondary CU traits than CP-only on dimensions characterized by harmony/emotionality, we performed exploratory analyses investigating whether parent-child synchrony characterized by behavioral coordination/cooperation between the three CP subgroups differed between types of play interaction (i.e., child-led, parent-led, cleanup task). These exploratory analyses were conducted because prior studies have found differences in synchrony and subsequent child outcomes depending on the type of play interaction (Krijnen et al., 2023). We found that relative to the primary CU group, mother-child dyads in the secondary CU group showed significantly less Coordinated Routines (*M*diff = -0.37, *p* = .042, 95% CI [-0.72, -0.01], *d* = 2.46) and Mutual Cooperation (*M*diff = -0.38, *p* = .010, 95% CI [-0.69, -0.07], *d* = 2.94) during the parent-led play interaction, but not during the child-led play or clean-up scenarios.

### Aim Two: Treatment-Related Change and Outcomes in Behavioral Synchrony

To test aim two, we used linear mixed modelling (LMM) with planned comparisons using the intent-to-treat sample (*n* = 175). We ran separate models for mothers and fathers, and separate models examining baseline to post-treatment (i.e., active treatment), and post-treatment to follow-up (i.e., maintenance of gains) outcomes. For each outcome variable, we estimated a baseline random intercept model, including timepoint (as a factor), CP group, and the interaction of time*group as fixed effects. We included treatment version and child age as covariates in all models. See Supplemental Tables 4 and 5 for LMM results.

Results of LMM indicated that, across groups, mother-child dyads showed significant improvements on all behavioral synchrony dimensions from baseline to post-treatment (*p* < .001), except for Emotional Ambience. The magnitude of improvement did not differ between the three CP subgroups for any mother-child synchrony dimension. Across groups, improvements in mother-child synchrony maintained to the three-month follow-up.

At post-treatment, the three CP subgroups did not significantly differ on any mother-child synchrony dimension. However, at the three-month follow-up, children classified as secondary CU (*M*diff = -1.03, *p* = .023, 95% CI [-1.92, -0.14], *d* = 2.28) and CP-only (*M*diff = -1.27, *p* = .014, 95% CI [-2.28, -0.26], *d* = 2.47) showed significantly lower Harmonious Communication than children classified as primary CU. Also, mother-child Emotional Ambience was significantly lower for CP-only than primary CU variants (*M*diff = -1.01, *p* = .044, 95% CI [-1.99, -0.03], *d* = 2.03) although the omnibus test was not significant (Supplemental Tables 6 and 7).

Father-child synchrony improved in Harmonious Communication and Emotional Ambience from baseline to post-treatment. Improvements in Mutual Cooperation were marginally significant, while father-child Coordinated Routines did not significantly improve. The magnitude of change from baseline to post-treatment did not differ between the three CP subgroups for any father-child synchrony dimension. Father-child Harmonious Communication deteriorated from post-treatment to three-month follow-up, with a significant group*time interaction and estimated marginal means indicating that this effect was driven by deterioration in the secondary CU group relative to further improvements in the primary CU group. While father-child Emotional Ambience did not significantly deteriorate from post-treatment to three-month follow-up, the group*time interaction was marginally significant. These results indicate a marginally significant difference in the magnitude of change between primary CU and secondary CU groups, such that the secondary CU group showed worsening father-child Emotional Ambience from post-treatment to three-month follow-up while the primary CU group improved. There was no significant deterioration in father-child Coordinated Routines or Mutual Cooperation from post-treatment to the follow-up.

The three CP subgroups did not significantly differ on any of the father-child synchrony scales at post-treatment. At the three-month follow-up, differences in Harmonious Communication were marginally significant between the primary CU and secondary CU groups (*M*diff = -1.15, *p* = .054, 95% CI [-2.33, 0.02], *d* = 1.94).

## Discussion

This study is the first to examine parent-child behavioral synchrony across well-established subgroups of clinic-referred young children with conduct problems (CP), and to evaluate treatment-related change in synchrony for both mother-child and father-child dyads. Three key findings emerged. First, at baseline, mother-child synchrony was significantly lower for children with elevated callous-unemotional (CU) traits, driven by the secondary CU subgroup. Second, following parent training involving an intensive relationship enhancement component (Leijten et al., 2018), synchrony improved broadly across dyads during active treatment. Third, at the three-month follow-up, mother-child synchrony remained lower for secondary CU variants (and CP-only on some indices) relative to primary CU variants. Father-child Harmonious Communication declined over follow-up in secondary CU relative to continued gains in primary CU variants. We discuss each finding in turn.

### Subgroup Differences in Baseline Synchrony

Lower mother-child synchrony in secondary CU traits, particularly in affective dimensions, converges with findings from community samples linking CU traits with reduced parent-child synchrony and warmth and extends them to a clinical, early childhood sample (Kochanska et al., 2013). This pattern is theoretically coherent: CU-related emotion processing difficulties, lower parental warmth, and higher parenting stress plausibly undermine mutual enjoyment and connected communication. Notably, baseline differences were not observed for fathers, and behavioral synchrony facets (Coordinated Routines, Mutual Cooperation) did not differ overall by CP subgroup. Exploratory task-level analyses suggested that, for mothers, secondary CU variants showed lower coordination (i.e., comfortable, shared understanding of procedural expectations) and cooperation (i.e., openness to each other’s influence, cooperation) compared to primary CU variants, specifically during parent-led play, but not child-led or clean-up. Given sample size constraints for fathers and the exploratory nature of task-specific effects, these findings warrant replication before drawing firm conclusions.

### Implications for Etiological Models

Our findings of lower baseline mother-child synchrony for children with secondary CU traits, relative to primary CU traits and CP-only, refine etiological models of secondary CU traits by identifying lower affective synchrony—reduced mutual enjoyment and smooth, connected exchanges—as an observable interaction context in early childhood. Within models positing heightened emotional sensitivity in secondary CU traits and greater exposure to adverse or rejecting parenting, recurrent low-synchrony interactions may contribute to cumulative stress in the dyad (Kimonis, 2023). Although causality cannot be inferred from the cross-sectional baseline contrasts, these data prioritize affective synchrony as a candidate mechanism to test longitudinally within etiological frameworks.

### Treatment-Related Change and Maintenance

Parent training (PCIT/PCIT-CU) was associated with improvements in parent-child synchrony during the active treatment phase for mothers (most dimensions) and fathers (Harmonious Communication, Emotional Ambience), consistent with interventions that coach in-the-moment responsiveness, positive attention, and contingent discipline (Fleming & Kimonis, 2018). However, subgroup differences re-emerged or widened by three months post-treatment: mother-child affective synchrony remained lower for secondary CU than primary CU variants, suggesting that these dyads struggled to have positive, back-and-forth communicative exchanges, particularly communication that promoted intimacy and connection. Father-child Harmonious Communication declined for secondary CU variants while improving for primary CU variants. Effects did not deteriorate uniformly (i.e., several mother-child gains maintained), but attenuation was most evident for secondary CU variants on affective synchrony facets. Overall, this pattern of results suggests that parent-child synchrony may be more difficult to maintain for secondary CU variants once treatment ends, aligning with other findings of deterioration in outcomes for secondary CU relative to primary CU variants after treatment ends (Fleming et al., 2023). These findings urge further consideration of potential factors contributing to fade-out effects for this most vulnerable subgroup.

### Clinical Implications

A plausible explanation for the suboptimal treatment outcomes for secondary CU dyads is that, while PCIT improves both parent and child behaviors (Fleming et al., 2022, 2025; Kimonis et al., 2019; Thomas et al., 2017), it may not as readily shift their feelings and perceptions of one another (Fleming, 2023; Fleming et al., 2025; Mah & Johnston, 2008). For instance, Harmonious Communication and Emotional Ambience reflect the degree to which interactions are a “source of pleasure for both” and promote “intimacy and connection” (Aksan et al., 2006). Our findings suggest that, while these facets improve for all dyads over active treatment, months later, parents and their children with secondary CU traits appear to enjoy their interactions less than other dyads. This reduced genuine and observable enjoyment may hint at insufficient and/or unsustained shifts in parental attitudes towards their children with secondary CU traits. While PCIT coaches parents in what to say and do, it has been criticized for inadequately targeting parents’ harsh or unhelpful cognitions and emotions about their child (Fleming et al., 2025). This represents an important area for future research. One direction may be to examine whether child factors such as anger-irritability, which are theorized to characterize secondary CU variants (Kimonis, 2023), contribute to maladaptive parental thoughts, emotions, and behaviours and whether emerging interventions targeting anger-irritability enhance treatment effects and their maintenance (Leibenluft et al., 2024).

Given its relevance to concurrent and future parent and child outcomes, synchrony in parent-child interactions should be a key focus of assessment and treatment for children with CP. Regarding assessment, clinicians should endeavor to observe how parents interact with their child across various contexts, as dyadic behaviors may differ between child-led and parent-led activities, for example. These insights can aid in identifying a dyad’s baseline level of mutual reciprocity, responsiveness, and enjoyment in their interactions, which are strong foundations for the parent-child relationship, to pinpoint specific parent and child behaviors to target through coaching. Some suggest that clinicians may benefit from regularly eliciting and assessing parents’ cognitions regarding their child’s behaviors (e.g., permanence or intentionality of the behaviors) and the parent-child relationship throughout treatment (Fleming et al., 2025), particularly among parents of children with secondary CU traits for whom parental cognitions are less warm and more harsh (Kaouar et al., 2023). This may involve encouraging parents to reflect on what they think and feel when their child misbehaves, to collaboratively identify and work through any cognitive biases that may hinder adaptive interactions with their child (Fleming et al., 2025).

### Study Limitations and Strengths

The present study provides important insights into dyadic synchrony for subgroups of children with CP, but it has some limitations. First, father participation was lower than mother participation, reducing power to detect subgroup differences in father-child synchrony. Second, observations used to code synchrony were derived from brief, standardized tasks that may not fully capture home interactions. Third, we did not have data from parent-child dyads of typically-developing children, which limited our capacity to understand how the patterns in our CP subgroups diverged. Fourth, treatment version (PCIT vs. PCIT-CU) varied within the longitudinal subsample; although analyses controlled for treatment type, differential effects by treatment version could not be fully tested. Study strengths include the clinical, early-childhood sample classified into established CP subgroups using well-validated measures; inclusion of both mothers and fathers; focus on discrete synchrony dimensions using a validated coding system; and a longitudinal study design capable of testing change and short-term maintenance. Within our longitudinal subsample, we had relatively low treatment attrition and between assessment time points. Our use of hierarchical linear modelling allowed us to statistically handle any remaining missingness in the data.

## Conclusions

In clinic-referred young children, secondary CU presentations are marked by lower affective facets of mother-child synchrony at baseline and show attenuated maintenance of synchrony gains following parent training. These findings refine etiological hypotheses by pinpointing affective synchrony as a salient dyadic process in secondary CU traits and suggest that sustaining improvements may require synchrony-focused augmentations and follow-up support tailored to CP subtype and caregiver dyad.

## Supplementary Information

Below is the link to the electronic supplementary material.

Supplementary Material 1

## Data Availability

This study was not a clinical trial (critical trial number not applicable). However, partial data from this study were drawn from clinical trials registered with the Australian and New Zealand Clinical Trials Registry (ACTRN1261600028040, registration date: 03 March 2016; ACTRN12616000221459, registration date: 18 February 2016). Treatment data used in this study are not publicly accessible, and analyses for this research were not pre-registered.

## Footnotes

1 These correlations were no longer significant after controlling for child CP severity (*r* = − .13, *p* = .057; *r* = − .12, *p* = .085, respectively).
