# Supplementary material for: Out of Sync: Parent-Child Behavioral Synchrony in Conduct Problem Subgroups
Source: Res Child Adolesc Psychopathol. 2026 Jul 30;54(4):96. doi: 10.1007/s10802-026-01491-6 (PMC13421254; doi:10.1007/s10802-026-01491-6)
Supplement: Supplementary file 1 — Supplementary Material 1 [file 10802_2026_1491_MOESM1_ESM.docx]

**Supplemental Methodological Materials**

**Present Study Sample Details**

Two trials in the present study oversampled children with elevated CU traits for research studies testing a targeted treatment for this subpopulation (Fleming & Kimonis, 2018; Kimonis et al., 2019). Oversampling of children with CU traits was achieved by advertising help for families in managing their preschooler’s difficult behaviors, including temper tantrums, disobedience, anger and irritability, low motivation, little remorse, little empathy, shallow emotions, and where discipline is ineffective.

**Group Classification Approach**

Children in this study were classified into three CP subgroups based on resolved scores (i.e., summing the highest item rating across reporters) on the 24-item *Inventory of Callous-Unemotional Traits* *- Preschool Version* (Kimonis et al., 2016), and the Internalizing composite scale of the *Achenbach System of Empirically Based Assessment -* *Child Behavior Checklist* (ASEBA CBCL; Achenbach & Rescorla, 2000, 2001). The established cut-off score of 64*T* on the CBCL Internalizing scale was used to identify clinically elevated internalizing problems. Because there is currently no universally accepted threshold for defining elevated CU traits in early childhood, we adopted a cut-off score of 29 on the ICU, based on its prior use in categorizing CP subgroups in young children (Kaouar et al., 2023) and its strong empirical grounding in older developmental samples. Specifically, this threshold has been derived using both empirical and normative methods to identify elevated CU traits in adolescent populations (Colins et al., 2018; Kemp et al., 2021). When benchmarked against community norms for preschool-aged children, this cut-off corresponds to approximately one standard deviation above the mean (Ezpeleta et al., 2013) and reflects an average item endorsement between “somewhat true” and “very true,” indicating a clinically meaningful elevation rather than a marginal increase in trait levels.

**Information on Missing Data**

For the present study, there were *n* = 12 families who attended the baseline assessment but did not complete key study measures needed to classify children into CP subgroups, and so they were excluded from this study. There were *n* = 2 families where both parents were mothers, and for these families, the biological mother was included in analyses. There were *n* = 2 families for whom only the father attended the assessment(s) and completed measures. Post-treatment and/or three-month follow-up data from *n* = 7 families were excluded due to the family dropping out of treatment prior to these assessments. Data from *n* = 1 family were excluded from longitudinal analyses (aim 2) as they completed a treatment other than PCIT or PCIT-CU, due to the developmental level of the child. Due to changes in the assessment protocol at the community clinic and/or interruptions on the day of assessment, not all parent-child dyads completed all three play scenarios during their assessment. Total numbers were as follows: *n* = 168 mothers, *n* = 99 fathers completed all three play scenarios; *n* = 28 mothers, *n* = 8 fathers completed only two scenarios, and *n* = 24 mothers completed only one play scenario. Scores were prorated to account for this missingness when calculating final synchrony scores.

**Attrition Analyses**

***Comparison of Assessment Attendees vs. Non-Attendees***

Parents who attended follow-up assessment(s) were compared to parents who did not attend these assessments on baseline, main study variables including child age, child sex, CP, CU traits, internalizing problems, and parent-child synchrony dimensions.

**Post-Treatment Assessment**. Groups did not significantly differ on any main study variables.

**Three-Month Follow-Up Assessment**. Children who attended the three-month follow-up assessment were significantly younger than children of mothers who did not attend this assessment. There were no group differences for fathers.

**Measures**

The following provides details regarding the administration, scoring, and established psychometric properties of each study measure.

***Conduct Problems*.** In the current study, *N* = 232 mothers and *n* = 124 fathers completed the ECBI at baseline, *n* = 87 mothers and *n* = 52 fathers at post-treatment, and *n* = 80 mothers and *n* = 53 fathers at three-month follow-up.

***CU Traits*.** The Preschool version of the ICU was adapted from the original Parent version to ensure the measure was developmentally appropriate for preschool-age children. Specifically, the original Item 3 (i.e., “Is concerned about schoolwork”) was replaced with “Seems motivated to do his/her best in structured activities.” In the current study, *N* = 232 mothers and *n* = 121 fathers completed the ICU at baseline, *n* = 81 mothers and *n* = 50 fathers at post-treatment, and *n* = 70 mothers and *n* = 49 fathers at three-month follow-up.

***Internalizing Symptoms*.** *­*In the current study, *N* = 230 mothers and *n* = 126 fathers completed the CBCL at baseline.

***Parent-Child Behavioral Synchrony.*** The *Mutually Responsive Orientation* scale (MRO; Aksan et al., 2006) was used to measure synchrony. For the purposes of the present study, a wider range of numerical anchor points were included for ratings of the four synchrony dimensions, to increase variability in scores. The final MRO coding sheet presented each of the four synchrony dimensions to be rated on a 5-point scale ranging from 1 to 3 (low = score of 1, low-medium = score of 1.5, medium = score of 2, medium-high = score of 2.5, high = score of 3). These scores were then qualitatively used to inform the Total synchrony score for each play scenario, which was rated on a 9-point scale ranging from 1-5, increasing in 0.5 increments (where 1 = very low synchrony/ poor relationship, and 5 = very high synchrony/ excellent relationship). For instance, according to the manual, a dyad displaying instances of mutual responsiveness and positive affect, and only rarely showing disconnectedness or negative affect, would be given a score of 4 (i.e., reasonable synchrony/ reasonable relationship). However, only the MRO dimension scores were used for this study, and not the Total score. Four of the coders were trained by the fifth master coder. Coders were introduced to the MRO coding system, and practiced coding videos until 80% reliability was achieved for each coder against the master coder. Subsequently, coders met fortnightly to code videos as a group, discuss difficult or tricky coding situations, and to prevent coder drift. To ensure coder masking, each video was assigned a random number using an electronic random number generator. MRO scores have shown moderate stability over time among toddlers, across both mother-child and father-child dyads (Aksan et al., 2006), with scores cohering substantially across different interaction situations (Kim et al., 2015).

**Supplemental Statistical Materials**

**Data Screening Procedure**

Prior to conducting main study analyses, data were screened for outliers and assumptions of planned analyses were tested. Residuals in our data were non-normally distributed according to Shapiro-Wilk tests, and so generalized linear models (GLM) were run to test baseline (cross-sectional) study aims. Any outlying values identified in our parent-reported data were retained in their raw form as these scores reflect true parent-reported values, and as such were not winsorized or transformed. For observed, researcher-rated synchrony scores, outliers exceeding ±3 *SD* were winsorized to either the 5^th^ or 95^th^ percentile, as appropriate. Only *n* = 1 mother’s post-treatment Coordinated Routines score was 3 *SD* below the mean, and was subsequently winsorized to the 5^th^ percentile.

**Supplemental Group Comparison Analyses**

Independent samples *t*-tests were conducted to compare younger (2-4 years) versus older children (5-7 years) on main study variables at baseline, to assess for potential differences between preschool-age versus school-age children. CP scores were significantly higher for older children (*M* = 176.10, *SD* = 26.38) than younger children (*M* = 164.13, *SD* = 33.06), *t*(232) = -2.82, *p* = .005, 95% CI [-20.34, -3.60], *d* = 0.39. CU trait scores were also significantly higher for older children (*M* = 36.17, *SD* = 10.42) than younger children (*M* = 31.72, *SD* = 10.59), *t*(232) = -3.08, *p* = .002, 95% CI [-7.30 -1.60], *d* = 0.42. There were no differences in Internalizing scores between age groups.

In terms of parent-child behavioral synchrony, mother-child Harmonious Communication scores were significantly lower for older children (*M* = 5.72, *SD* = 1.28) than younger children (*M* = 6.39, *SD* = 1.75), *t*(217) = 2.93, *p* = .004, 95% CI [0.22, 1.12], *d* = 0.42. Mother-child Emotional Ambience scores were also significantly lower for older children (*M* = 6.44, *SD* = 1.45) than younger children (*M* = 6.99, *SD* = 1.59), *t*(218) = 2.49, *p* = .014, 95% CI [0.11, 0.98], *d* = 0.35. There were no significant differences for father-child synchrony scores between age groups.

We also conducted independent samples *t*-tests to compare boys and girls on main study variables. CP scores were higher for boys (*M* = 171.10, *SD* = 29.73) than girls (*M* = 160.07, *SD* = 34.69), *t*(232) = 2.37, *p* = .019, 95% CI [1.87, 20.20], *d* = 0.36. CU trait scores were also higher for boys (*M* = 34.30, *SD* = 10.32) than girls (*M* = 30.26, *SD* = 11.36), *t*(232) = 2.54, *p* = .012, 95% CI [0.91, 7.16], *d* = 0.38. There were no gender differences in child Internalizing scores. In terms of parent-child synchrony, only one significant difference emerged: Father-child Mutual Cooperation scores were significantly lower for boys (*M* = 6.62, *SD* = 1.60) than girls (*M* = 7.48, *SD* = 1.19), *t*(105) = -2.54, *p* = .013, 95% CI [-1.54, -0.19], *d* = 0.57.

Planned comparisons were also conducted to test group differences between the three CP subgroups on child age, sex, and selected main study measures. There were no significant sex differences between the three CP subgroups according to Bonferroni-adjusted chi-square tests (*p > .*008). Children with high CU traits (i.e., primary CU and secondary CU variants) were significantly older than children with CP-only, *F*(1, 232) = 14.51, *p* < .001, η_p_^2^ = .06. This is likely due to children being significantly older at the university clinic that intentionally oversampled for children with elevated CU traits (*F*(1, 232) = 109.35, *p* < .001, η_p_^2^ = .32), than the community clinic. CP scores were highest for secondary CU compared to primary CU variants (*F*(1, 231) = 4.80, *p* = .029, η_p_^2^ = .02), and the CP-only group (*F*(1, 231) = 79.97, *p* < .001, η_p_^2^ = .26), and higher for primary CU variants than the CP-only group (*F*(1, 231) = 32.85, *p* < .001, η_p_^2^ = .13). Holding CP constant, CU trait scores were higher for secondary CU variants compared to CP-only (*F*(1, 230) = 161.88, *p* < .001, η_p_^2^ = .41), and higher for primary CU variants than CP-only (*F*(1, 230) = 124.74, *p* < .001, η_p_^2^ = .35). Lastly, holding CP constant, Internalizing scores were highest for secondary CU compared to primary CU variants (*F*(1, 230) = 122.19, *p* < .001, η_p_^2^ = .35), and the CP-only group (*F*(1, 230) = 46.95, *p* < .001, η_p_^2^ = .17), and higher for the CP-only group than for primary CU variants (*F*(1, 230) = 11.04, *p* = .001, η_p_^2^ = .05).

There were no significant differences between mother-child and father-child synchrony across MRO dimension scores at baseline (p > .05). There were no significant CP group differences in the number of treatment sessions (i.e., dose) completed by the family (*p* > .05), nor in the groups’ treatment drop-out versus completion rates (*p > .*008 for Bonferroni-adjusted chi-square tests).

Baseline, main study analyses were repeated controlling for child age and sex. Results did not differ when including age or sex as covariates (results available upon request). Thus, given child age differences on many main study variables, child age was retained as a covariate in baseline analyses, alongside child CP scores, to provide the most parsimonious account of the data.

**Supplemental Figure 1**

*Participant Flow Chart*


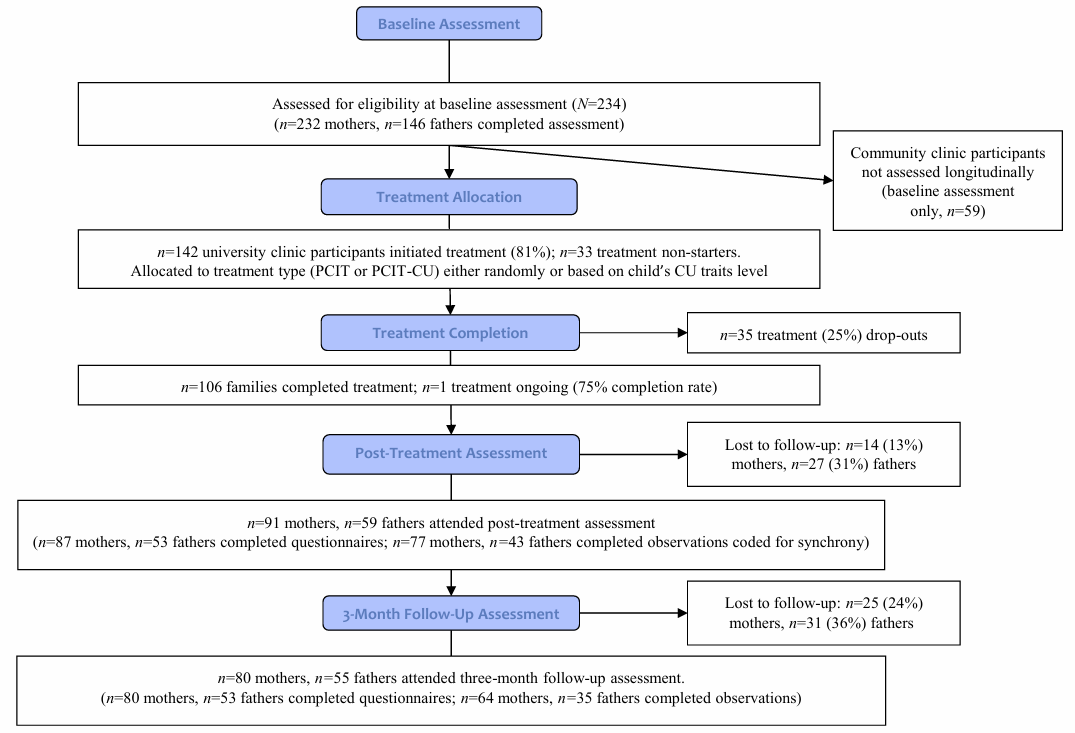


**Supplemental Table 1**

*Child demographics and descriptive statistics for selected study measures across clinics*

| Clinic | Age  (*SD*) | Sex  (*n*) | ECBI  (*SD*) | ICU  (*SD*) | CBCL INT  (*SD*) |
| --- | --- | --- | --- | --- | --- |
| University (*n* = 175) | 4.99  (1.39) | 131 boys  44 girls | 173.92  (26.68) | 34.96  (10.35) | 64.81  (9.76) |
| *Range* | 1.98-8.54 | -- | 90-238 | 4-71 | 34-88 |
| Community (*n* = 59) | 3.04  (0.64) | 43 boys  16 girls | 151.50  (37.90) | 28.21  (10.28) | 63.92  (9.27) |
| *Range* | 2.00-4.50 | -- | 52-227 | 4-56 | 37-79 |
| Total (*N* = 234) | 4.50  (1.50) | 174 boys  60 girls | 168.27  (31.38) | 33.26  (10.72) | 64.59  (9.62) |

*Note.* ECBI = Eyberg Child Behavior Inventory; ICU = Inventory of Callous-Unemotional Traits; CBCL INT = Child Behavior Checklist Internalizing. Mean resolved scores are presented for baseline ECBI (raw Intensity total), ICU (raw total), and CBCL (*T*-scores) measures. Mean age given in years

**Supplemental Table 2**

*Mutually Responsive Orientation (MRO) scale dimensions*

| MRO Dimension | Dimension Description and Examples |
| --- | --- |
| Coordinated  Routines | Extent to which the dyad displays coordinated activity and settles comfortably into routine activities that become scripted over time. For example:   - **Low**: Routines are a source of conflict, or are very choppy and rough - **High**: Implicit shared procedural expectations |
| Harmonious Communication | Extent to which verbal and nonverbal aspects of communication flow smoothly. For example:   - **Low**: Participates in very little or no communication. - **High**: Communication flows effortlessly. Exchanges promote intimacy and connection |
| Mutual  Cooperation | Extent to which the dyad effectively resolves potential sources of conflict and are open to each other’s influence. For example:   - **Low**: Dyad is unable to accept roles. Frequent resistance. - **High**: Subtle influences are sufficient for cooperation. |
| Emotional  Ambience | Extent to which the dyad enjoys an emotionally positive atmosphere indicating clear pleasure in each other’s company. For example:   - **Low**: Negative ambience permeates interaction. Positive affect basically absent. - **High**: Natural displays of affection which are a source of pleasure for both. |

*Note*. Adapted from Aksan et al. (2006) *Mutually Responsive Orientation* coding manual

**Supplemental Table 3**

*Means for CP subgroups on mothers’ and fathers’ baseline parent-child behavioral synchrony scores*

| Mother-Child | CP only  (*n* = 69) | Primary  (*n* = 55) | Secondary  (*n* = 96) | Wald χ² | *df* | *p-*value |
| --- | --- | --- | --- | --- | --- | --- |
| Coordinated Routines | 7.10  (0.23) | 6.67  (0.23) | 6.53  (0.18) | 3.41 | 2 | .182 |
| Harmonious Communication | 6.62^a^  (0.22) | 6.09^ab^  (0.22) | 5.87^b^  (0.17) | 6.29 | 2 | .043 |
| Mutual  Cooperation | 7.03  (0.22) | 6.70  (0.22) | 6.50  (0.18) | 3.09 | 2 | .213 |
| Emotional Ambience | 7.28^a^  (0.21) | 6.60^ab^  (0.21) | 6.57^b^  (0.17) | 7.24 | 2 | .027 |
| Father-Child | CP only  (*n* = 18) | Primary  (*n* = 33) | Secondary  (*n* = 56) | Wald χ² | *df* | *p-*value |
| Coordinated Routines | 7.07  (0.41) | 6.76  (0.29) | 6.77  (0.22) | 0.46 | 2 | .794 |
| Harmonious Communication | 6.31  (0.35) | 6.22  (0.24) | 5.78  (0.19) | 2.83 | 2 | .243 |
| Mutual  Cooperation | 7.07  (0.37) | 7.03  (0.26) | 6.63  (0.20) | 1.94 | 2 | .378 |
| Emotional Ambience | 6.86  (0.38) | 6.92  (0.27) | 6.53  (0.21) | 1.49 | 2 | .475 |

*Note*. Estimated marginal means (*SE*); different superscripts (^a, b^) denote Bonferroni-adjusted significant differences between groups in pair-wise comparisons. Primary = high CU traits and low INT; Secondary = high CU traits and high INT. Wald χ², *p*-value, and *df* represent tests of CP group as a model predictor. Child age and resolved CP severity entered as covariates for all analyses

**Aim One: Subgroup Differences in Baseline Parent-Child Behavioral Synchrony**

We ran further analyses for Aim 1, additionally controlling for child Attention-Deficit/ Hyperactivity Disorder (ADHD) symptoms, measured using the Attention-Deficit/Hyperactivity Problems scale of the ASEBA CBCL.

When comparing children high versus low on CU traits, mother-child dyads in the two groups did not differ in their Coordinated Routines (*M*diff = -0.51, *p* = .076, 95% CI [-1.08, 0.05]) or Mutual Cooperation scores (*M*diff = -0.43, *p* = .121, 95% CI [-0.98, 0.11]). However, children with CP+CU had significantly lower mother-child Harmonious Communication (*M*diff = -0.62, *p* = .024, 95% CI [-1.15, -0.08]) and Emotional Ambience (*M*diff = -0.69, *p* = .009, 95% CI [-1.20, -0.17]) than children with CP-only, when controlling for CP intensity. There were no significant differences in father-child synchrony scores between children with high versus low levels of CU traits.

Planned comparisons between the three CP subgroups showed that mother-child interactions for the secondary CU group were characterized by marginally lower Harmonious Communication (*M*diff = -0.69, *p* = .068, 95% CI [-1.41, 0.04]) and significantly lower Emotional Ambience (*M*diff = -0.69, *p* = .050, 95% CI [-1.38, 0.00]), than the CP-only group. There were no other group differences for the remaining mother-child synchrony dimensions. For father-child dyads, there were no significant differences between CP subgroups on any synchrony dimensions.

**Supplemental Table 4**

Results of linear mixed modelling examining baseline to post-treatment changes in parent-child synchrony

| Variable | Time |  | Time*Group |  |
| --- | --- | --- | --- | --- |
| Mothers | β [95% CI] | *p* | β [95% CI] | *p* |
| Coordinated Routines | -1.13 [-1.70, -0.55] | <.001 |  |  |
| CP v SCU |  |  | 0.37 [-0.62, 1.35] | .462 |
| PCU v SCU |  |  | 0.23 [-0.75, 1.21] | .640 |
| Harmonious Communication | -1.02 [-1.54, -0.50] | <.001 |  |  |
| CP v SCU |  |  | -0.16 [-1.05, 0.73] | .721 |
| PCU v SCU |  |  | 0.23 [-0.65, 1.11] | .608 |
| Mutual Cooperation | -0.94 [-1.46, -0.41] | <.001 |  |  |
| CP v SCU |  |  | 0.02 [-0.88, 0.92] | .965 |
| PCU v SCU |  |  | 0.36 [-0.53, 1.25] | .420 |
| Emotional Ambience | -0.43 [-0.92, 0.07] | .089 |  |  |
| CP v SCU |  |  | 0.31 [-0.53, 1.15] | .465 |
| PCU v SCU |  |  | 0.06 [-0.78, 0.90] | .891 |
| Fathers | β [95% CI] | *p* | β [95% CI] | *p* |
| Coordinated Routines | -0.68 [-1.52, 0.16] | .111 |  |  |
| CP v SCU |  |  | -0.17 [-1.73, 1.39] | .825 |
| PCU v SCU |  |  | 0.24 [-1.17, 1.64] | .740 |
| Harmonious Communication | -1.29 [-1.96, -0.62] | <.001 |  |  |
| CP v SCU |  |  | 0.04 [-1.21, 1.29] | .950 |
| PCU v SCU |  |  | 1.06 [-0.07, 2.18] | .066 |
| Mutual Cooperation | -0.74 [-1.48, 0.00] | .051 |  |  |
| CP v SCU |  |  | -0.73 [-2.11, 0.65] | .293 |
| PCU v SCU |  |  | 0.55 [-0.69, 1.79] | .377 |
| Emotional Ambience | -0.94 [-1.69, -0.18] | .016 |  |  |
| CP v SCU |  |  | -0.16 [-1.56, 1.25] | .823 |
| PCU v SCU |  |  | 1.08 [-0.19, 2.34] | .094 |

*Note*. PCU = Primary CU; SCU = Secondary CU; Time = baseline vs. post-treatment; Group = participant classification by CP subgroup as CP-only vs. PCU vs. SCU; Tx = treatment classified as PCIT vs. PCIT-CU

**Supplemental Table 5**

*Results of linear mixed modelling examining post-treatment to three-month follow-up changes in parent-child synchrony*

| Variable | Time |  | Time*Group |  |
| --- | --- | --- | --- | --- |
| Mothers | β [95% CI] | *p* | β [95% CI] | *p* |
| Coordinated Routines | 0.20 [-0.46, 0.87] | .547 |  |  |
| CP v SCU |  |  | -0.35 [-1.48, 0.78] | .540 |
| PCU v SCU |  |  | -0.51 [-1.72, 0.70] | .409 |
| Harmonious Communication | -0.09 [-0.68, 0.49] | .755 |  |  |
| CP v SCU |  |  | 0.12 [-0.88, 1.12] | .807 |
| PCU v SCU |  |  | -0.83 [-1.91, 0.25] | .130 |
| Mutual Cooperation | -0.19 [-0.78, 0.40] | .520 |  |  |
| CP v SCU |  |  | 0.22 [-0.79, 1.22] | .669 |
| PCU v SCU |  |  | -0.46 [-1.54, 0.63] | .407 |
| Emotional Ambience | -0.21 [-0.76, 0.34] | .443 |  |  |
| CP v SCU |  |  | 0.13 [-0.81, 1.06] | .792 |
| PCU v SCU |  |  | -0.52 [-1.53, 0.50] | .314 |
| Fathers | β [95% CI] | *p* | β [95% CI] | *p* |
| Coordinated Routines | 0.45 [-0.43, 1.33] | .315 |  |  |
| CP v SCU |  |  | 0.09 [-1.54, 1.73] | .910 |
| PCU v SCU |  |  | -0.56 [-2.24, 1.12] | .508 |
| Harmonious Communication | 1.00 [0.21, 1.78] | .013 |  |  |
| CP v SCU |  |  | -0.37 [-1.82, 1.09] | .621 |
| PCU v SCU |  |  | -1.56 [-3.05, -0.08] | .039 |
| Mutual Cooperation | 0.49 [-0.31, 1.29] | .223 |  |  |
| CP v SCU |  |  | 0.49 [-0.99, 1.97] | .512 |
| PCU v SCU |  |  | -0.68 [-2.21, 0.84] | .375 |
| Emotional Ambience | 0.77 [-0.10, 1.65] | .083 |  |  |
| CP v SCU |  |  | 0.12 [-1.51, 1.75] | .887 |
| PCU v SCU |  |  | -1.61 [-3.25, 0.02] | .053 |

*Note*. PCU = Primary CU; SCU = Secondary CU; Time = post-treatment vs. three-month follow-up; Group = participant classification by CP subgroup as CP-only vs. PCU vs. SCU

**Supplemental Table 6**

*Means for CP subgroups on mothers’ and fathers’ post-treatment parent-child behavioral synchrony scores*

| Mother-Child | CP only  (*n* = 50) | Primary  (*n* = 58) | Secondary  (*n* = 106) | *F* value | *p-*value | *df* |
| --- | --- | --- | --- | --- | --- | --- |
| Coordinated  Routines | 7.31  (0.33) | 7.82  (0.34) | 7.83  (0.25) | 0.87 | .421 | 2 |
| Harmonious Communication | 6.74  (0.31) | 7.07  (0.32) | 6.88  (0.23) | 0.28 | .755 | 2 |
| Mutual  Cooperation | 7.32  (0.32) | 7.57  (0.33) | 7.45  (0.24) | 0.15 | .864 | 2 |
| Emotional  Ambience | 6.83  (0.31) | 7.24  (0.32) | 6.98  (0.23) | 0.42 | .659 | 2 |
| Father-Child | CP only  (*n* = 23) | Primary  (*n* = 38) | Secondary  (*n* = 68) | *F* value | *p-*value | *df* |
| Coordinated  Routines | 7.66  (0.50) | 7.39  (0.49) | 7.55  (0.36) | 0.08 | .926 | 2 |
| Harmonious Communication | 7.10  (0.39) | 6.61  (0.38) | 7.14  (0.28) | 0.67 | .513 | 2 |
| Mutual  Cooperation | 8.01  (0.44) | 7.38  (0.43) | 7.39  (0.31) | 0.74 | .481 | 2 |
| Emotional  Ambience | 7.62  (0.44) | 6.92  (0.43) | 7.42  (0.31) | 0.69 | .502 | 2 |

*Note*. Estimated marginal means (*SE*); different superscripts (^a, b^) denote significant differences between groups in pair-wise comparisons. Primary = high CU traits and low INT; Secondary = high CU traits and high INT. *F* value, *p*-value, and *df* represent statistics for omnibus tests of group differences. Child age and treatment type entered as covariates for all analyses

**Supplemental Table 7**

*Means for CP subgroups on mothers’ and fathers’ three-month follow-up parent-child behavioral synchrony scores*

| Mother-Child | CP only  (*n* = 67) | Primary  (*n* = 71) | Secondary  (*n* = 140) | *F* value | *p-*value | *df* |
| --- | --- | --- | --- | --- | --- | --- |
| Coordinated  Routines | 7.47  (0.36) | 8.11  (0.41) | 7.63  (0.25) | 0.74 | .478 | 2 |
| Harmonious Communication | 6.75^a^  (0.34) | 8.02^b^  (0.38) | 6.99^a^  (0.24) | 3.49 | .032 | 2 |
| Mutual  Cooperation | 7.31  (0.34) | 8.20  (0.38) | 7.65  (0.24) | 1.52 | .221 | 2 |
| Emotional  Ambience | 6.95^a^  (0.33) | 7.96^b^  (0.37) | 7.22^ab^  (0.23) | 2.19 | .114 | 2 |
| Father-Child | CP only  (*n* = 30) | Primary  (*n* = 45) | Secondary  (*n* = 89) | *F* value | *p-*value | *df* |
| Coordinated  Routines | 7.20  (0.62) | 7.50  (0.60) | 7.02  (0.35) | 0.24 | .784 | 2 |
| Harmonious Communication | 6.56  (0.53) | 7.24  (0.52) | 6.09  (0.30) | 1.93 | .148 | 2 |
| Mutual  Cooperation | 7.11  (0.57) | 7.54  (0.55) | 6.79  (0.32) | 0.73 | .484 | 2 |
| Emotional  Ambience | 6.82  (0.57) | 7.75  (0.56) | 6.63  (0.32) | 1.54 | .219 | 2 |

*Note*. Estimated marginal means (*SE*); different superscripts (^a, b^) denote significant differences between groups in pair-wise comparisons. Primary = high CU traits and low INT; Secondary = high CU traits and high INT. *F* value, *p*-value, and *df* represent statistics for omnibus tests of group differences. Child age and treatment type entered as covariates for all analyses

**Supplemental Figures 2.**

*Line graphs depicting estimated marginal means for each synchrony dimension, across CP subgroups and timepoints*

*Note*. Mean mother-child Coordinated Routines for each CP group, at each timepoint. Covariates include child CP and age at baseline, and child age and treatment type at post-treatment and follow-up

*Note*. Mean mother-child Mutual Cooperation for each CP group, at each timepoint. Covariates include child CP and age at baseline, and child age and treatment type at post-treatment and follow-up

*Note*. Mean mother-child Harmonious Communication for each CP group, at each timepoint. Covariates include child CP and age at baseline, and child age and treatment type at post-treatment and follow-up

*Note*. Mean mother-child Emotional Ambience for each CP group, at each timepoint. Covariates include child CP and age at baseline, and child age and treatment type at post-treatment and follow-up

**Supplemental References**

Achenbach, T. M., & Rescorla, L. A. (2000; 2001). *Manual for the ASEBA Preschool/ School-Age Forms & Profiles*. University of Vermont Research Center for Children, Youth, & Families.

Aksan, N., Kochanska, G., & Ortmann, M. R. (2006). Mutually responsive orientation between parents and their young children: Toward methodological advances in the science of relationships. *Developmental Psychology*, *42*(5), 833-848. <https://doi.org/10.1037/0012-1649.42.5.833>

Colins, O. F., Andershed, H., Salekin, R. T., & Fanti, K. A. (2018). Comparing different approaches for subtyping children with conduct problems: Callous-unemotional traits only versus the multidimensional psychopathy construct. *Journal of Psychopathology and Behavioral Assessment*, *40*(1), 6-15. <https://doi.org/10.1007/s10862-018-9653-y>

Ezpeleta, L., Osa, N. D. L., Granero, R., Penelo, E., & Domènech, J. M. (2013). Inventory of Callous-Unemotional Traits in a community sample of preschoolers. *Journal of Clinical Child and Adolescent Psychology*, *42*(1), 91-105.<https://doi.org/10.1080/15374416.2012.734221>

Fleming, G. E., & Kimonis, E. R. (2018). PCIT for Children with Callous-Unemotional Traits. In L. N. Niec (Ed.), *Handbook of Parent-Child Interaction Therapy: Innovations and applications for research and practice* (pp. 19-34). Springer.

Kaouar, S., Fleming, G. E., Neo, B., Hawes, D. J., Eapen, V., & Kimonis, E. R. (2023). Dimensions of warm parenting attributions differentiate conduct problem subtypes in young children. *Research on Child and Adolescent Psychopathology*, *52*(2), 223-236. <https://doi.org/10.1007/s10802-023-01111-7>

Kemp, E. C., Frick, P. J., Matlasz, T. M., Clark, J. E., Robertson, E. L., Ray, J. V., … Cauffman, E. (2021). Developing cutoff scores for the Inventory of Callous-Unemotional Traits (ICU) in justice-involved and community samples. *Journal of Clinical Child and Adolescent Psychology.* Advance online publication. <https://doi.org/10.1080/15374416.2021.1955371>

Kim, S., Boldt, L. J., & Kochanska, G. (2015). From parent-child mutuality to security to socialization outcomes: Developmental cascade toward positive adaptation in preadolescence. *Attachment & Human Development*, *17*(5), 472-491. <https://doi.org/10.1080/14616734.2015.1072832>

Kimonis, E. R., Fanti, K. A., Anastassiou-Hadjicharalambous, X., Mertan, B., Goulter, N., & Katsimicha, E. (2016). Can callous-unemotional traits be reliably measured in preschoolers? *Journal of Abnormal Child Psychology*, *44*(4), 625-638. <https://doi.org/10.1007/s10802-015-0075-y>

Kimonis, E. R., Fleming, G., Briggs, N., Brouwer-French, L., Frick, P. J., Hawes, D. J., Bagner, D. M., Thomas, R., & Dadds, M. (2019). Parent-Child Interaction Therapy adapted for preschoolers with callous-unemotional traits: An open trial pilot study. *Journal of Clinical Child & Adolescent Psychology*, *48*(sup1), S347-S361. <https://doi.org/10.1080/15374416.2018.1479966>
